# Selected Immunological Mediators and Cervical Microbial Signatures in Women with Chlamydia trachomatis Infection

**DOI:** 10.1128/mSystems.00094-19

**Published:** 2019-06-04

**Authors:** Simone Filardo, Marisa Di Pietro, Giulia Tranquilli, Maria Agnese Latino, Nadia Recine, Maria Grazia Porpora, Rosa Sessa

**Affiliations:** a Section of Microbiology, Department of Public Health and Infectious Diseases, University of Rome “Sapienza,” Rome, Italy; b Unit of Bacteriology, STIs Diagnostic Centre, Sant’Anna Hospital, Turin, Italy; c Department of Gynaecology, Obstetrics and Urology, University of Rome “Sapienza,” Rome, Italy; University of Massachusetts Dartmouth

**Keywords:** 16S rRNA gene, cervical microbiota, *Chlamydia trachomatis*, cytokines, lactoferrin

## Abstract

In the female genital ecosystem, the complex interplay between the host immune system and the resident microflora protects against urogenital pathogens, like Chlamydia trachomatis. C. trachomatis is responsible for urethritis and cervicitis; however, most chlamydial infections are asymptomatic and, thus, not treated, potentially leading to severe reproductive sequelae. Here we investigated the interaction between the levels of selected immune mediators and the community state types of the cervical microbiota in C. trachomatis-infected women. Cervical samples from 42 C. trachomatis-positive women and 103 matched healthy controls were analyzed through the metagenomic analysis of the hypervariable region v4 of the 16S rRNA gene and the determination of lactoferrin, interleukin 1α (IL-1α), IL-6, alpha interferon (IFN-α), IFN-β, and IFN-γ by ELISA. Overall, C. trachomatis infection was significantly associated with a microbiota dominated by anaerobic bacteria (*P* = 0.000002). In addition, a network of Gardnerella vaginalis, Prevotella amnii, Prevotella buccalis, Prevotella timonensis, Aerococcus christensenii, and Variovorax guangxiensis has been identified as a potential biomarker of C. trachomatis infection through multiple statistical approaches. Again, chlamydial infection was significantly correlated with an increased production of lactoferrin, IL-6, IL-1α, IFN-α, and IFN-β (*P* < 0.05), whereas very low levels of IFN-γ were observed in C. trachomatis-infected women, levels similar to those detected in healthy women. Our findings show a distinctive signature of C. trachomatis genital infection, characterized by a specific bacterial network, constituted by anaerobes, as well as by increased levels of lactoferrin and proinflammatory cytokines (IL-1α, IL-6, IFN-α, and IFN-β), accompanied by low levels of IFN-γ.

**IMPORTANCE** To our knowledge, this is the first study that investigated the association of C. trachomatis with the cervical levels of lactoferrin and selected inflammatory mediators and their correlation with the different community state types characterizing the female genital ecosystem. C. trachomatis, known as the leading cause of bacterial sexually transmitted diseases, continues to be an important public health problem worldwide for its increasing incidence and the risk of developing severe reproductive sequelae, like pelvic inflammatory disease and infertility. Specifically, C. trachomatis tend to persist in the female genital tract, leading to a chronic inflammatory state characterized by increased production of immune mediators responsible for tissue damage. Therefore, our study may help to broaden the knowledge on the complex interplay between the female genital microbiota and the host immune system in response to C. trachomatis infection.

## Abstract (precis)

To our knowledge, this is the first study that investigated the association of C. trachomatis with the cervical levels of lactoferrin and selected inflammatory mediators and their correlation with the different community state types characterizing the female genital ecosystem. C. trachomatis, known as the leading cause of bacterial sexually transmitted diseases, continues to be an important public health problem worldwide for its increasing incidence and the risk of developing severe reproductive sequelae, like pelvic inflammatory disease and infertility. Specifically, C. trachomatis tend to persist in the female genital tract, leading to a chronic inflammatory state characterized by increased production of immune mediators responsible for tissue damage. Therefore, our study may help to broaden the knowledge on the complex interplay between the female genital microbiota and the host immune system in response to C. trachomatis infection.

## INTRODUCTION

In the female genital ecosystem, the protection against infectious agents is due to the epithelial barrier, continuously lubricated by the cervicovaginal fluid (CVF) and populated by numerous microorganisms that exist in a regulated mutual relationship with the host (1, 2).

The genital epithelium, in addition to acting as a physical barrier, triggers the production of proinflammatory cytokines, like interleukin 1α (IL-1α), IL-6, and interferons and hence contributes to the host immune response toward pathogens (3). Similarly, the CVF contains several host defense compounds, including lactoferrin, that has recently acquired importance for its extensive biological effects (4). For example, lactoferrin has been demonstrated to possess antibacterial activity by inhibiting the growth and dissemination of pathogens through different mechanisms, like iron sequestration or the inhibition of bacterial adhesion to the host cells, as evidenced in Pseudomonas aeruginosa and adherent Escherichia coli HB101, respectively (4).

The cervicovaginal microbiota in healthy women, mostly populated by *Lactobacillus* spp., is widely considered an important line of defense against urogenital pathogens (1, 5, 6). In recent years, culture-independent high-resolution techniques based on the analysis of 16S rRNA gene sequences have contributed to expand our knowledge on the composition of the genital microbiota, leading to its classification into five community state types (CSTs) (6, 7). CSTs I, II, III, and V are dominated by Lactobacillus crispatus, Lactobacillus gasseri, Lactobacillus iners, and Lactobacillus jensenii, respectively, whereas CST-IV is characterized by a reduced number of *Lactobacillus* spp. and an increased diversity of anaerobic bacterial species, including Gardnerella vaginalis, Atopobium vaginae, *Prevotella* spp., etc. (7). CST-IV is often associated with the condition of dysbiosis, known to increase the risk of acquiring sexually transmitted diseases (STDs), including Chlamydia trachomatis (8, 9).

C. trachomatis is the leading cause of bacterial STDs, with more than 130 million new cases per year, according to the most recent World Health Organization’s estimates (10), and it is responsible for urethritis, cervicitis, and salpingitis. However, most genital infections in women are asymptomatic and, thus, not treated, potentially leading to severe reproductive sequelae, such as pelvic inflammatory disease (PID), ectopic pregnancy, and obstructive infertility (11, 12). In fact, it is estimated that 40 to 60% of cases of PID as well as two-thirds of all cases of tubal factor infertility and one-third of all cases of ectopic pregnancy are most likely due to asymptomatic C. trachomatis infection (12).

C. trachomatis genital infection tends to persist in the host, leading to increased production of genital immune mediators, including IL-6, IL-1α, and gamma interferon (IFN-γ), responsible for the inflammatory damage underlying the development of severe sequelae (12, 13).

In a previous pilot study, we showed that women with C. trachomatis genital infection possessed a heterogeneous microbiota with increased species richness and diversity, dominated by anaerobes (14). Herein, we sought to define the different CSTs associated with C. trachomatis infection and to correlate them with the levels of selected immune mediators to investigate their complex interaction in the female genital ecosystem. To do so, we performed the metagenomic analysis of sequenced 16S rRNA gene amplicons with the Illumina MiSeq sequencer, and we analyzed the host defense mechanisms through the detection of lactoferrin, IL-1α, IL-6, IFN-α, IFN-β, and IFN-γ in the CVF.

## RESULTS

### Study population characteristics.

One hundred forty-five women of reproductive age were enrolled in the present study, 42 women positive for C. trachomatis and 103 matched healthy controls.

All 145 samples underwent the 16S rRNA amplicon-based microbiome analysis, and 7 samples were excluded from further processing due to a total number of reads of <100. Population characteristics are summarized in Table 1. A statistically significant association was observed between C. trachomatis infection and high-risk sexual behaviors as well as bacterial vaginosis (*P* < 0.05).

**TABLE 1:** Study population characteristics

Characteristic or risk factor^*a*^	% women (no. of women [*n*])^*b*^		*P* value^*c*^
	C. trachomatis-positive women (*n* = 39)	Healthy women (*n* = 99)	
Age, yr [median (IQR)]	24 (10.5)	25 (13)	NS

Risk factors			
Smoking	0.00 (0)	0.00 (0)	NS
High-risk sexual behavior	43.59 (17)	15.15 (15)	0.00058
First intercourse at 16 years	20.51 (8)	6.06 (6)	0.016
New partner in the last 6 mo	33.33 (13)	10.10 (10)	0.0017
Multiple partners	30.77 (12)	9.09 (9)	0.0024
No barrier contraception	89.74 (35)	75.76 (75)	NS
Past STI	23.08 (9)	20.20 (20)	NS
Bacterial vaginosis (Nugent score, 7–10)	20.51 (8)	7.07 ( 7)	0.029

a IQR, interquartile range; STI, sexually transmitted infection.

b Percentage of women with the indicated risk factor (number of women with the risk factor) unless indicated otherwise.

c NS, not significant.

### Error rate estimation via mock microbial community cosequencing.

To assess the error rate of the MiSeq Illumina sequencing, we cosequenced a mock microbial community of known composition that contained eight different bacterial species. The types and relative abundances of the bacteria present in the mock community, as well as the species and relative abundances identified by sequencing, are shown in Table 2. The estimated error rate was 0.0314%.

**TABLE 2:** Theoretical and sequenced composition of the mock community

Bacterial species	Sequencing result (%)	Theoretical^*a*^ mock community composition (%)
Lactobacillus fermentum	22.82	18.8
Salmonella enterica	17.23	11.3
Bacillus subtilis	14.26	15.7
Escherichia coli	11.23	10
Staphylococcus aureus	10.92	13.3
Listeria monocytogenes	10.40	15.9
Pseudomonas aeruginosa	8.11	4.6
Enterococcus faecalis	5.03	10.4

a The theoretical composition was calculated from genomic DNA by the following formula: 16S copy number = [total genomic DNA (g) × unit conversion constant (bp/g)]/[genome size (bp) × 16S copy number per genome].

### Composition of the cervical microbiota in C. trachomatis-positive women and healthy controls.

**FIG 1:**
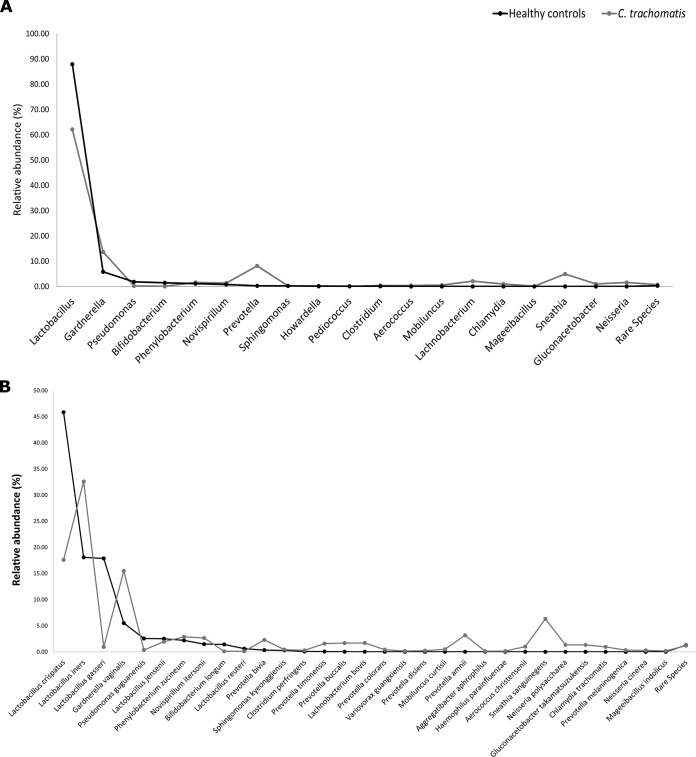
Cervical microbial composition in C. trachomatis-positive and healthy women at the genus level (A) and species level (B). Only taxa with abundances greater than 0.01% in any sample were included in the graphs.

The cervical microbiota from healthy controls was dominated by Lactobacillus crispatus (mean relative abundance, 45.8% [standard error of the mean {SEM}, 4.5]), followed by L. iners (18.1% [33.5]) and L. gasseri (17.9% [3.1]) (Fig. 1B; see also Fig. S1 in the supplemental material).

10.1128/mSystems.00094-19.1 FIG S1 Heatmap indicating the changes at the species level of the cervical microbial composition in C. trachomatis-positive women and healthy controls. The legend below the heatmap represents each participant sample ID and genital health status. The relative abundance of bacteria in each species is indicated by a color gradient from black (low abundance) to white (high abundance). Download FIG S1, TIF file, 2.5 MB. Copyright © 2019 Filardo et al. 2019 Filardo et al. This content is distributed under the terms of the Creative Commons Attribution 4.0 International license.

In C. trachomatis-positive women, the cervical microbiota was characterized by a decrease in L. crispatus (17.6% [5.1]; *P* < 0.01) and L. gasseri (0.9% [0.9]; *P* < 0.001) (Fig. 1A and B), whereas *L. iners* (32.6% [6]; *P* < 0.0001) was far more prevalent alongside anaerobic bacteria, like Gardnerella vaginalis (15.5% [3.5] versus 5.5% [1.8]; *P* < 0.000001), Prevotella amnii (3.2% [1.2] versus 0.001% [0.001]; *P* < 0.05), Prevotella buccalis (1.7% [0.7] versus 0.004% [0.002]; *P* < 0.01), Prevotella timonensis (1.6% [0.7] versus 0.05% [0.04]; *P* < 0.01) and Aerococcus christensenii (1% [0.7] versus 0.001% [0.001]; *P* < 0.0001) (Fig. 1B and Fig. S1).

More than 50% of healthy women had CST-I, and a minority of them had CST-II and CST-III microbiota. In contrast, a higher number of C. trachomatis-positive women had CST-III microbiota than healthy controls, even though it did not reach statistical significance (*P* = 0.054). More interestingly, the highest proportion of C. trachomatis-positive women had a CST-IV microbiota, mostly driven by *G. vaginalis* and *Prevotella* spp. (*P* < 0.00001). Overall, C. trachomatis infection was significantly associated with a combination of CST-III and CST-IV microbiota (74% versus 26% in healthy controls; *P* < 0.000001) (Table 3 and Fig. S2).

**TABLE 3:** Community state types in C. trachomatis-positive and healthy women

Community state type^*a*^	% of women (no. of women [*n*])		*P* value
	C. trachomatis-positive women (*n* = 39)	Healthy women (*n* = 99)	
CST-I (L. crispatus)	25.64 (10)	56.57 (56)	0.0011
CST-II (L. gasseri)	0.00 (0)	17.17 (17)	0.0032
CST-III (L. iners)	35.89 (14)	20.20 (20)	0.054
CST-IV (anaerobic bacteria)	38.46 (15)	6.06 ( 6)	0.000002

a CST, community state type.

10.1128/mSystems.00094-19.2 FIG S2 Cluster dendrogram representing the community state types of the cervical microbiota in C. trachomatis-infected and healthy women. CSTs were assigned by Ward’s linkage hierarchical clustering based on the Jensen-Shannon divergence between all pairs of community states. To determine the best number of clusters, the Silhouette measure of the degree of confidence was calculated for each *k* number of clusters, where *k* was between 2 and 10. The maximum Silhouette value index was at *k* = 4, and hence, the dendrogram was cut at a level that had four leaves. Download FIG S2, TIF file, 1.3 MB. Copyright © 2019 Filardo et al. 2019 Filardo et al. This content is distributed under the terms of the Creative Commons Attribution 4.0 International license.

The presence of genital symptoms in women with C. trachomatis infection was not related to a specific CST (Table 4).

**TABLE 4:** Prevalence of CSTs in C. trachomatis-positive women in relation to genital symptoms

Community state type^*a*^	% C. trachomatis-positive women (no. of women [*n*]) (*n* = 39)		*P* value
	Asymptomatic	Symptomatic	
CST-I	21.05 (4)	20.00 (4)	0.94
CST-III	31.58 (6)	40.00 (8)	0.58
CST-IV	47.37 (9)	40.00 (8)	0.64

a CST, community state type.

### Increased microbial species diversity and richness in C. trachomatis-positive women.

**FIG 2:**
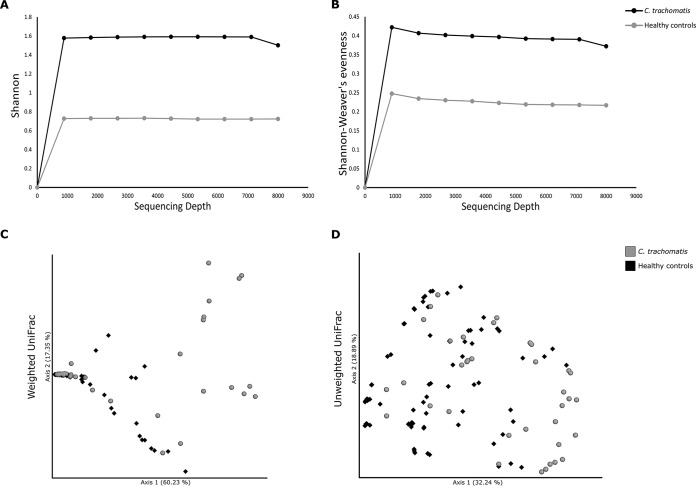
Alpha and beta diversity of cervical microbiota in C. trachomatis-positive and healthy women. Rarefaction curves of Shannon’s diversity index (A) and Shannon-Weaver’s evenness (B), used as measures of alpha diversity within groups. Principal coordinate analysis (PCoA) of weighted (C) and unweighted (D) UniFrac analyses, used as measures of beta diversity between groups.

Initially, we characterized the diversity and richness of the microbial species populating the cervical microbiota in either C. trachomatis-positive or healthy women, determining the alpha diversity via Shannon’s diversity and Shannon-Weaver’s evenness indexes that consider both the abundance and evenness of the bacterial species. Both indexes showed that C. trachomatis infection was significantly associated with increased heterogeneity of the bacterial species (Fig. 2A and B; *P* < 0.0001).

Then, we investigated the diversity in microbial communities between C. trachomatis-positive and healthy women, calculating the beta diversity via the weighted and unweighted UniFrac analyses, the first based on sequence distances in the phylogenetic tree and on their relative abundances, whereas the latter was based solely on the sequence distances. Both analyses showed a statistically significant separation of the cervical microbiota from the two groups (Fig. 2C and D; *P* = 0.001).

### Specific taxa associated with C. trachomatis infection.

To identify a biomarker for C. trachomatis infection in our metagenomic data, we utilized two different approaches, namely, the linear discriminant analysis (LDA) coupled with effect size measurement (LEfSe) (15) and the analysis of composition of microbiomes (ANCOM) (16).

**FIG 3:**
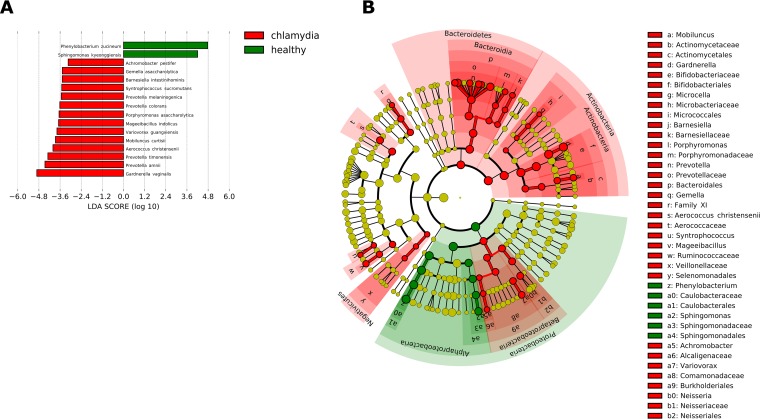
Linear discriminant analysis with effect size measurement (LEfSe) on the cervical microbial composition in C. trachomatis*-*positive women compared to healthy controls. (A) Histogram of the LDA scores computed for statistically significant differentially abundant taxa between C. trachomatis-positive women and healthy controls. (B) Cladogram highlighting the relationship of significantly different taxa between C. trachomatis*-*positive women and healthy controls. Differences are represented in the color of the most abundant class, and each circle’s diameter is proportional to the taxon’s abundance.

The LEfSe analysis identified 14 bacterial species, including *A. christensenii*, *P. timonensis*, *P. amnii,* and *G. vaginalis*, as strongly associated with C. trachomatis infection (LDA score of >4.0) (Fig. 3).

The ANCOM test revealed *L. iners*, *P. amnii*, *P. timonensis*, *P. buccalis*, *A. christensenii*, *S. sanguinegens*, Variovorax guangxiensis, and *G. vaginalis* as strongly associated with C. trachomatis infection, whereas only L. gasseri was associated with the cervical microbiota in healthy controls (Table 5).

**TABLE 5:** ANCOM analysis in C. trachomatis-positive and healthy women

Species	% of women with the indicated species (SEM)		*W*^*a*^	*F*^*b*^
	C. trachomatis-positive women	Healthy women		
Gardnerella vaginalis	15.5 (3.5)	5.5 (1.8)	123	33.1
Prevotella amnii	3.2 (1.2)	0.0013 (0.0014)	114	25.1
Prevotella buccalis	1.7 (0.7)	0.004 (0.002)	118	32.7
Prevotella timonensis	1.6 (0.7)	0.05 (0.04)	116	30.5
Aerococcus christensenii	1 (0.7)	0.001 (0.001)	116	42.2
Lactobacillus gasseri	0.9 (0.6)	17.9 (3.08)	127	17.1
Lactobacillus iners	32.6 (6.02)	18.1 (3.5)	115	20.7
Sneathia sanguinegens	6.3 (2.3)	0 (0)	122	54.3
Variovorax guangxiensis	0.15 (0.05)	0.003 (0.002)	116	68.5

a *W* statistics represent the number of times the null hypothesis is rejected for a given taxon.

b *F* statistics represent a measure of how different a group is from the average for a specific taxon.

**FIG 4:**
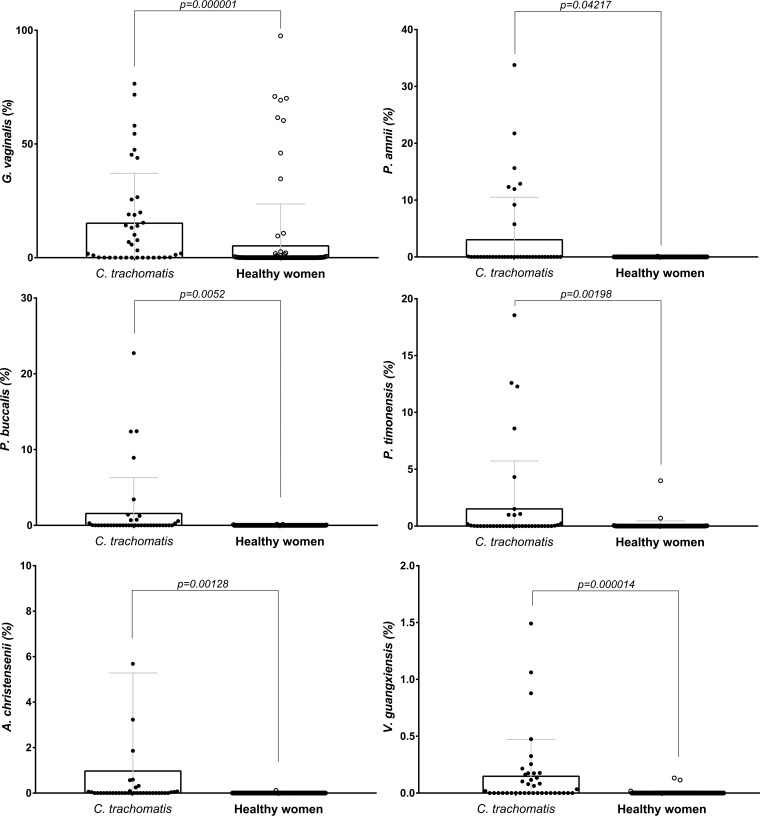
Comparison of the relative abundances of the bacterial species identified by both LEfSe and ANCOM analyses in C. trachomatis-positive and healthy women.

The relative abundances of the key phylotypes successfully differentiated in both analyses (*G. vaginalis*, *P. amnii*, *P. buccalis*, *P. timonensis*, *A. christensenii*, and *V. guangxiensis*) were further compared between the two groups, and all of them were significantly associated with C. trachomatis infection (Fig. 4).

### A distinct microbial network associated with C. trachomatis infection.

To identify correlations between taxonomic units within the microbial communities of C. trachomatis-positive women and healthy controls, we performed the sparse correlations for compositional data (SparCC) analysis, retaining only correlations with a |*r|* of >0.35, as previously described (17).

**FIG 5:**
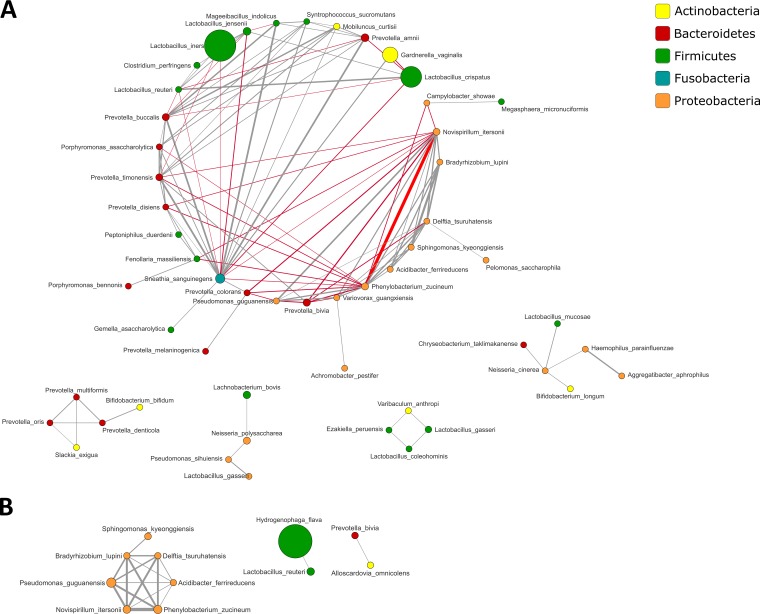
Cooccurrence analysis of bacterial taxa in the cervical microbiota from C. trachomatis-infected (A) and healthy (B) women. Nodes represent operational taxonomic units (OTUs) with size reflecting the OTU’s average fraction in the community, and colors indicate the phylum to which the OTU belongs. Edges between nodes represent correlations between the nodes they connect, with the edge’s thickness indicating the correlation magnitude, and both positive (gray) and negative (red) associations shown. Only edges corresponding to correlation whose magnitude is greater than 0.35 are drawn. Unconnected nodes are omitted.

Overall, a higher number of correlations and, thus, a more complex bacterial network was found in C. trachomatis-positive women than in healthy controls (Fig. 5 and Fig. S3).

10.1128/mSystems.00094-19.3 FIG S3 Cooccurrence analysis of bacterial taxa in the cervical microbiota from C. trachomatis-infected (A) and healthy (B) women. Areas of the network that are strongly interconnected are represented as separate modules. Nodes represent OTUs with size reflecting the OTU’s average fraction in the community, and colors correspond to the phylum to which the OTU belongs. Edges between nodes represent correlations between the nodes they connect, with the edge’s thickness indicating the correlation magnitude. Only positive correlations are used for finding modules of cooccurring OTUs, and only correlations whose magnitude is greater than 0.35 are included. Unconnected nodes are omitted. Download FIG S3, TIF file, 2.7 MB. Copyright © 2019 Filardo et al. 2019 Filardo et al. This content is distributed under the terms of the Creative Commons Attribution 4.0 International license.

Specifically, the cervical microbiota of women with C. trachomatis infection was characterized by positive correlations within bacteria associated with dysbiosis, like *Prevotella* spp., *Porphyromonas* spp., and *G. vaginalis*, whereas strong negative correlations were observed between these species and *Lactobacillus* spp., including L. crispatus, L. iners, and L. jensenii.

### Increased lactoferrin and proinflammatory cytokine levels in C. trachomatis-positive women.

**FIG 6:**
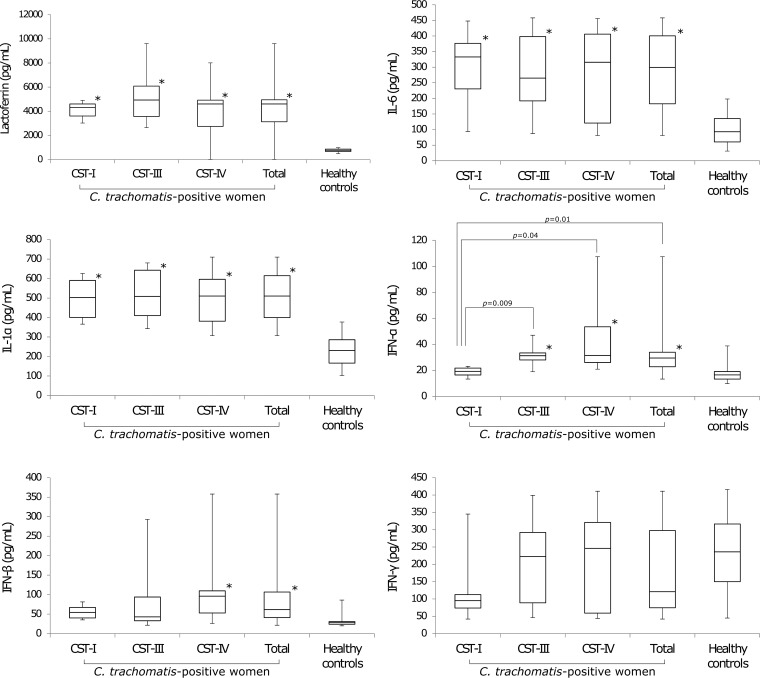
Lactoferrin and proinflammatory cytokine levels in C. trachomatis*-*positive and healthy women. Values that are significantly different from the values from healthy controls (*P* < 0.05) are indicated (*).

We found significantly increased levels of lactoferrin in CVF samples from women infected by C. trachomatis compared to healthy controls (Fig. 6); however, no association was found with a specific CST.

Concerning the proinflammatory cytokines, the levels of IL-1α and IL-6 were significantly higher in the CVF samples from C. trachomatis*-*positive women than healthy controls. Similarly, significantly increased levels of IFN-α and IFN-β were observed in *Chlamydia*-infected women, whereas no statistically significant difference was found for IFN-γ between the two groups. Overall, no CST in C. trachomatis-positive women was associated with high or low levels of IL-1α, IL-6, IFN-β, and IFN-γ. However, significantly lower levels of IFN-α were observed in C. trachomatis*-*infected women with CST-I compared to those with CST-III and CST-IV microbiota (Fig. 6).

No significant association was observed between the levels of lactoferrin or proinflammatory cytokines and genital symptoms in C. trachomatis-positive women (Table 6).

**TABLE 6:** Lactoferrin and proinflammatory cytokine levels related to genital symptoms in C. trachomatis-positive women

Lactoferrin or cytokine	Level in C. trachomatis-positive women (*n* = 39)^*a*^		*P* value
	Asymptomatic	Symptomatic	
Lactoferrin (ng/ml)	4,262.4 ± 297.9	4,783.9 ± 512.6	0.39
IL-6 (pg/ml)	256.2 ± 28.7	303.1 ± 30.1	0.27
IL-1α (pg/ml)	487.9 ± 28.2	526.2 ± 29.2	0.35
IFN-α (pg/ml)	32.1 ± 5.5	36.2 ± 5.9	0.32
IFN-β (pg/ml)	84.3 ± 17.3	84.2 ± 20.6	0.57
IFN-γ (pg/ml)	164.2 ± 27.5	206.0 ± 29.6	0.31

a Lactoferrin and cytokine levels are expressed as means ± SEM.

Last, in healthy women, lactoferrin, IL-1α, IL-6, IFN-α, IFN-β, and IFN-γ were consistently low, independent of the composition of the cervical microbiota (Fig. S4).

10.1128/mSystems.00094-19.4 FIG S4 Lactoferrin and proinflammatory cytokine levels in healthy controls in relation to the CSTs. C. trachomatis*-*positive women are reported as a comparison. Download FIG S4, TIF file, 1.8 MB. Copyright © 2019 Filardo et al. 2019 Filardo et al. This content is distributed under the terms of the Creative Commons Attribution 4.0 International license.

## DISCUSSION

This is the first study investigating the association of C. trachomatis with the cervical levels of lactoferrin and proinflammatory cytokines and their correlation with the different CSTs characterizing the female genital ecosystem.

The main findings of our study follow: (i) correlation of C. trachomatis infection with a cervical microbiota categorized CST-IV (predominance of anaerobic bacteria) (*P* = 0.000002); (ii) identification of a specific bacterial network, characterized by *G. vaginalis*, *P. amnii*, *P. buccalis*, *P. timonensis*, *A. christensenii*, and *V. guangxiensis*, as a potential biomarker of C. trachomatis infection (*P* < 0.05); (iii) association of C. trachomatis infection with significantly increased production of lactoferrin, IL-6, IL-1α, IFN-α, and IFN-β (*P* < 0.01); (iv) detection of very low levels of IFN-γ in C. trachomatis-positive women.

Concerning the cervical microbiota, our findings add to the notion that the presence of C. trachomatis is associated with a varied microbiota mostly dominated by anaerobes, including *G. vaginalis* and *Prevotella* spp., as evidenced by the prevalence of CST-IV in our patients. Interestingly, the combination of two statistical methods for biomarker discovery (LEfSe and ANCOM) revealed the association between C. trachomatis and *G. vaginalis*, the most predominant species, as well as *P. amnii*, *P. buccalis*, *P. timonensis*, *A. christensenii*, and *V. guangxiensis*. More importantly, through the statistical model for building correlation networks SparCC (17), we observed a strong cooccurrence within the above-mentioned bacterial species, suggesting the compelling hypothesis of a specific bacterial network as a biomarker for C. trachomatis infection; in fact, such a bacterial network is an intriguing hypothesis that may better describe the peculiar genital microenvironment associated with C. trachomatis.

Alongside the resident microflora, the female genital tract possesses other defense systems that protect against infectious threats. Among them, lactoferrin, synthesized and released in the CVF by mucosal epithelial cells and neutrophils, has recently been shown to modulate the host immune response and to exert antimicrobial activity against different pathogens (4).

Here we observed a higher production of lactoferrin in C. trachomatis-positive women than in healthy controls. This is not surprising, since increased levels of lactoferrin in the genital secretions of women with other STDs have been reported (18, 19). Moreover, in previous studies, we demonstrated the protective role of lactoferrin against C. trachomatis infection by inhibiting its adhesion and invasion to cervical epithelial cells (20, 21).

A more interesting finding of our study shows that cervical lactoferrin levels were not influenced by a specific CST, suggesting that the production of lactoferrin may most likely be elicited by the inflammatory response following C. trachomatis infection; this is further supported by studies showing the recruitment of lactoferrin-producing neutrophils to the site of infection (21).

In addition to lactoferrin, the host genital defense systems include a plethora of immune cells that release a wide array of cytokines in the extracellular space once a pathogen is detected (3).

Our findings show the ability of C. trachomatis to induce a distinct inflammatory state in the cervical microenvironment through the activation of Th1 and Th2 responses, as evidenced by the high levels of IL-1α, IL-6, IFN-α, and IFN-β determined in the CVF samples from chlamydia-infected women. Surprisingly, the levels of IFN-γ in the CVF samples from our patients were very low, like those observed in healthy women.

IL-1α is a Th2-type cytokine that contributes to the amplification of the inflammatory process following C. trachomatis infection (21–23), eliciting the production of other proinflammatory cytokines, like IL-6, and recruiting innate immune cells (23, 24). Simultaneously, tissue-resident macrophages and dendritic cells release type I interferons, namely, IFN-α and IFN-β (25). These cytokines, once considered only antiviral agents, mediate numerous antibacterial and immunoregulatory effects, including the regulation of IL-12 expression, that, in turn, induces the activation of natural killer cells as well as the development of Th1 CD4^+^ T cells (25). Subsequently, the immunoregulatory activity of type I interferons leads to the production of IFN-γ, a critical cytokine of the innate and adaptive immune defenses against obligate intracellular pathogens like C. trachomatis (26, 27). Indeed, IFN-γ is considered an antichlamydial agent for its ability to reduce tryptophan availability through the upregulation of the enzyme indoleamine 2,3-dioxygenase that metabolizes tryptophan into kynurenine, thus inhibiting chlamydia intracellular replication (28). Interestingly, evidence in the literature showed that the high kynurenine-to-tryptophan ratio as well as high levels of IFN-α and IFN-β decreased IFN-γ production by altering T-helper cell activity or negatively affecting IL-12 production and, hence, downregulating the immune response to infectious agents (25, 29).

In this regard, in our patients, the observation of low levels of IFN-γ, as well as high levels of IFN-α and IFN-β, leads to the hypothesis that increased production of type I interferons may be a potential mechanism underlying IFN-γ downregulation. However, we cannot exclude the possibility that an increase in the kynurenine-to-tryptophan ratio may also be responsible for decreased IFN-γ levels (29), hinting at complex regulatory pathways in response to C. trachomatis infection.

It should be noted that the low levels of cervical IFN-γ observed in our patients may also favor the development of chlamydial persistence, which is well-known to elicit a chronic inflammatory state, worsening, *de facto*, the clinical course of the infection and leading to severe reproductive sequelae (12, 13).

Interestingly, among all the cytokines observed in our patients, only IFN-α was influenced by the composition of the cervical microbiota in C. trachomatis-positive women, as evidenced by lower levels of this cytokine in women with CST-I than in women with CST-III or CST-IV microbiota (*P* < 0.05). This suggests that a cervical microbiota dominated by L. crispatus (CST-I) might possess anti-inflammatory properties, in accordance with other studies that observed increased inflammation in the absence of L. crispatus (30, 31). Nevertheless, the determination of other proinflammatory cytokines (IL-8, IL-12, tumor necrosis factor alpha [TNF-α]) will be required to fully characterize the host immune response to C. trachomatis in association with the different CSTs.

The main strengths of our study are the sequencing error estimation and the selection of a population whose size was estimated *a priori* to attain a statistical power of at least 80% and a level of significance of 5%. Furthermore, we enrolled only women with well-characterized healthy genital conditions in the control group in this study. In fact, all the participants positive for any genital pathogen other than C. trachomatis were excluded from the study, since an altered cervical microbiota has also been associated with other pathogens. Last, we excluded women with behavioral factors (antibiotic, probiotic, and/or prebiotic treatments) that could affect the composition of the cervical microbiota.

The selection of an appropriate number of participants and the application of stringent inclusion criteria allowed us to greatly diminish the impact of confounding bias related to the selection of the study population and to increase the probability of observing clinically significant differences between the groups, although the case-control nature of our study limited the possibility of discovering a causal relationship.

In conclusion, our findings show a distinctive signature of C. trachomatis genital infection, characterized by a specific bacterial network and by increased levels of lactoferrin and proinflammatory cytokines (IL-1α, IL-6, IFN-α, and IFN-β), accompanied by low levels of IFN-γ. This complex picture may have physiopathological relevance, since it might be responsible for the incomplete clearance of C. trachomatis, leading to a persistent infection and, hence, to the development of chronic reproductive sequelae.

In the future, it will be important to monitor the changes in the cervical microbiota as well as in the host immune responses before and after exposure to C. trachomatis to clarify their temporal dynamics.

## MATERIALS AND METHODS

### Study design and sample collection.

From January to July 2017, 42 women of Italian origin positive for C. trachomatis genital infection were selected among the patients attending the STIs Diagnostic Centre, Sant’Anna Hospital, Turin, Italy, and the Department of Gynaecology, Obstetrics and Urology at the University of Rome “Sapienza” for regular check-ups. For the control group, 103 racially and age-matched healthy women were recruited.

Women with sexual activity in the week prior to sampling, recent or current antibiotic and/or hormonal medications (oral or topical), as well as use of probiotics and/or prebiotics, were excluded from the study.

From each woman, two endocervical swabs (FLOQ swabs, Copan) were collected. One swab was used for C. trachomatis, Neisseria gonorrhoeae, Trichomonas vaginalis, *Mycoplasma*, *Candida*, human papillomavirus (HPV), and herpes simplex virus 2 (HSV-2) testing as previously described (32) and for the metagenomic analysis. The second swab was used for lactoferrin, IL-1α, IL-6, IFN-α, IFN-β, and IFN-γ determination. Cervical samples were taken 7 days after menses withdrawal and were immediately stored at −80°C until further processing.

All study participants were also examined for bacterial vaginosis (BV) and for the presence of symptoms. BV was assessed using Amsel criteria and confirmed using Gram stain criteria (Nugent score).

All study participants gave their written informed consent prior to sampling and provided a detailed personal, medical, and gynecological history. This study design and protocol was approved by the Umberto I University Hospital ethical committee (reference number 367/16) and conducted according to the principles expressed in the Declaration of Helsinki.

### DNA isolation and next-generation sequencing.

DNA isolation from endocervical swabs and the next-generation sequencing were performed as previously described (14). The primers 515F (5′-GTGCCAGCMGCCGCGGTAA-3′) and 806R (5′-GGACTACHVGGGTWTCTAAT-3′; Illumina Inc., USA) were utilized for the amplification of the V4 hypervariable region of the 16S rRNA gene.

### Sequencing data analysis.

To analyze the amplicon sequence data obtained from the MiSeq Illumina sequencer, we followed the standard operating procedure (SOP) developed for the mothur software package (version 1.39.5) (33) by Kozich et al. (34) and illustrated on the mothur website (https://www.mothur.org/wiki/MiSeq_SOP), accessed in January 2018.

The microbial community of each sample was assigned to a specific CST by following the approach described by Gajer et al. (7).

Shannon’s diversity and Shannon-Weaver’s evenness indexes were used as metrics for alpha diversity rarefaction analysis, whereas unweighted and weighted UniFrac distance matrixes were used as beta diversity measures, as previously described (35).

Differential taxonomic units between groups were identified using the LEfSe and ANCOM analyses as previously described (15, 16).

To identify correlations between taxonomic units within microbial communities, we performed the SparCC analysis as described by Friedman and Alm (17).

### Lactoferrin and proinflammatory cytokine determination.

Lactoferrin, IL-1α, IL-6, IFN-α, IFN-β, and IFN-γ levels were assayed in 99 out of 145 women enrolled because the sampling material was inadequate in 46 individuals.

The levels of lactoferrin, IL-1α, IL-6, IFN-α, IFN-β, and IFN-γ were determined by using the respective ELISA kits (Abcam, USA; BioVendor, Czech Republic; MyBioSource, Inc., USA), according to the manufacturer’s instructions.

### Statistical analysis.

Relative abundances of taxa were expressed as means ± standard error of means (SEM). Nonparametric *t* test based on Monte Carlo permutations was used for alpha diversity comparisons, and Adonis was used for category comparisons of distance matrixes, all calculated in QIIME 2 (36). The relative abundances of the taxa successfully differentiated in both LEfSe and ANCOM were compared between the groups using unpaired Wilcoxon rank sum test, considering ties among values.

Cytokine and lactoferrin levels were expressed as means ± standard deviations (SD) for at least two replicates. One-way analysis of variance (ANOVA) for multiple comparisons followed by pairwise *t* test for equal variances or Games-Howell *post hoc* test for unequal variances between groups were used for comparison of means. Bonferroni correction was used to correct for multiple hypothesis testing when needed. The chi-squared test was used for assessment of association of frequencies among groups (Fisher’s exact test was used when any cell had expected values of <5). The single or multiple inference significance level was set at 5%.

### Data availability.

Raw sequences were deposited into the NCBI’s Sequence Read Archive (SRA) under accession number PRJNA509578.
