# Supplementary figures and images for: Selected Immunological Mediators and Cervical Microbial Signatures in Women with Chlamydia trachomatis Infection
Source: mSystems. 2019 Jun 4;4(4):e00094-19. doi: 10.1128/mSystems.00094-19 (PMC6550367; doi:10.1128/mSystems.00094-19)

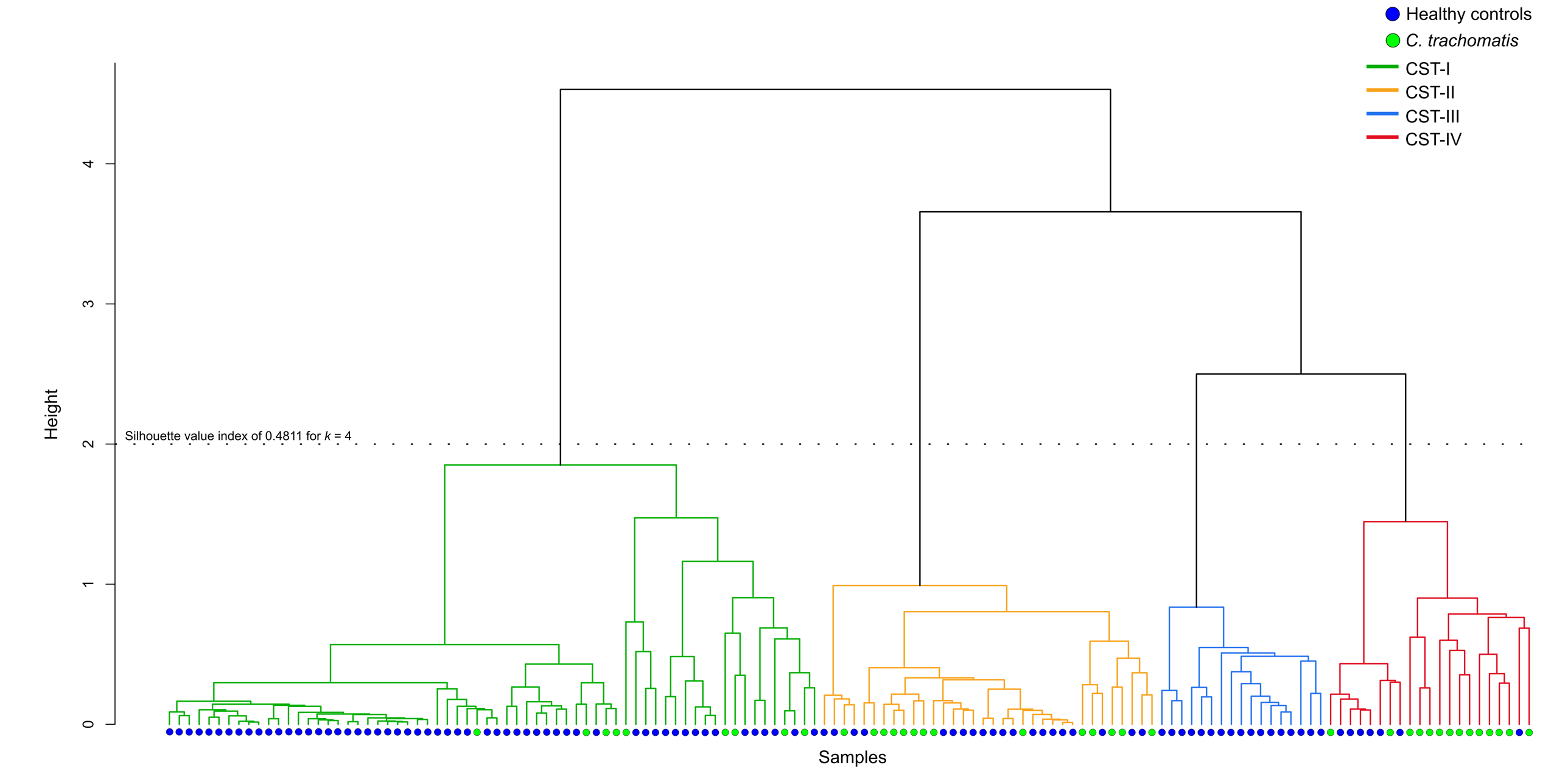

Supplement: FIG S2 [file mSystems.00094-19-sf002.tif]

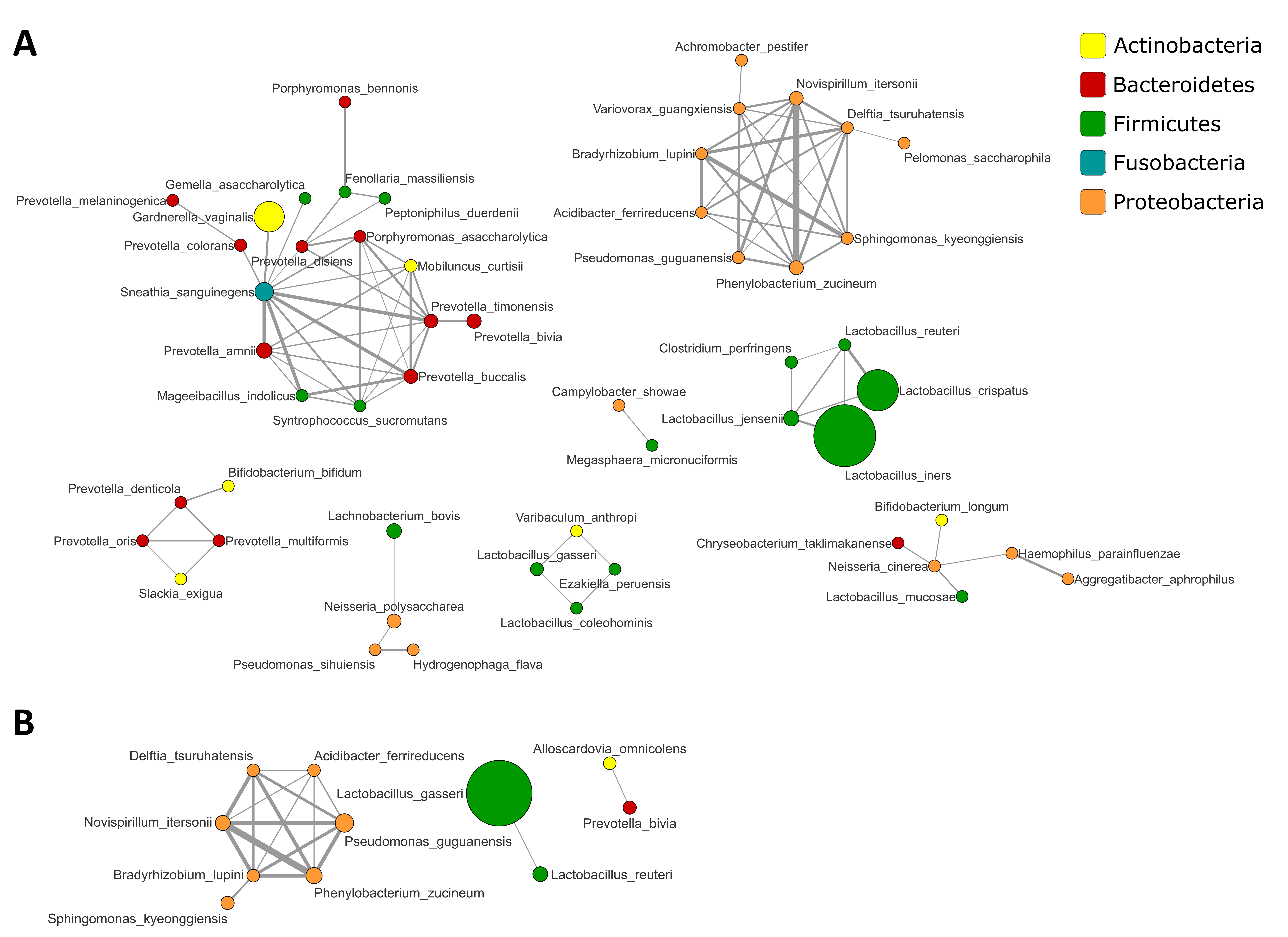

Supplement: FIG S3 [file mSystems.00094-19-sf003.tif]
